# Reduction of complications in femoral neck fracture fixation using femoral neck system: the importance of pre-compression technique and power bar position

**DOI:** 10.3389/fmed.2025.1677846

**Published:** 2025-11-03

**Authors:** Bin Chen, Dongze Lin, Fengfei Lin, Wenge Liu

**Affiliations:** ^1^Fujian Medical University Union Hospital, Fuzhou, Fujian, China; ^2^Fuzhou Second General Hospital, Fuzhou, Fujian, China; ^3^The Clinical Medical College of Fujian Medical University, Fuzhou, Fujian, China; ^4^Fujian Provincial Clinical Medical Research Center for First Aid and Rehabilitation in Orthopedic Trauma, Fuzhou, Fujian, China

**Keywords:** femoral neck fracture, fracture fixation, internal, complication, femoral neck system

## Abstract

**Background:**

To explore the causes of early- and mid-term complications and failures following femoral neck system (FNS) treatment for femoral neck fractures in young and middle-aged adults, to identify prevention and treatment strategies.

**Method:**

A retrospective analysis was conducted on the clinical data of 89 young and middle-aged adults patients with femoral neck fractures who received FNS treatment at our hospital between September 2019 and November 2021. Cases with early- and mid-term complications or failures were classified, and the potential causes and corresponding treatment measures were analyzed.

**Result:**

A total of 12 patients (13.5%) experienced complications, including femoral neck shortening in 9 cases (10.1%), lateral femoral cutaneous nerve injury in 1 case (1.1%), and internal fixation failure in 2 cases (2.2%). Univariable and multivariable analyses identified tip-apex distance (TAD) ≥ 25 mm, medial cortical comminution, poor reduction quality (Garden grades III–IV), and non-inferior power bar position as significant risk factors for complications. Patients treated with the pre-compression technique demonstrated a lower incidence of femoral neck shortening (0% vs. 17.6%) and significantly better hip function scores at 6-month and final follow-up compared to those without pre-compression.

**Conclusion:**

Strict adherence to operational standards, proper surgical techniques, improved fracture reduction quality, and appropriate weight-bearing control can help reduce early- and mid-term complications following FNS fixation for femoral neck fractures in young and middle-aged adults. The use of the pre-compression technique for the power bar, along with its placement in the lower to middle part of the femoral neck, may reduce femoral neck shortening and the risk of internal fixation cut-out.

## 1 Introduction

Femoral neck fractures are a common and severe traumatic condition in young and middle-aged adult's patients. The treatment goal is to maximize the restoration of hip function, prevent long-term complications such as femoral head necrosis, non-union, or malunion, and ultimately reduce the economic burden on society and families (1). However, femoral neck fractures in young and middle-aged adults present unique clinical challenges. These fractures are often complex due to high-energy trauma, the anatomically vulnerable blood supply to the femoral head, and high shear stresses. Additionally, the high activity levels of young and middle-aged adults and their significant demand for functional recovery further complicate treatment, making it a considerable challenge (2). Despite the wide range of surgical internal fixation options available through modern medical advancements (3), the incidence of femoral head necrosis remains as high as 14% (4). Biomechanical experiments and clinical studies have shown that conventional internal fixation techniques, such as hollow screws (CCS) and dynamic hip screws (DHS), have notable limitations in clinical applications. While CCS offers minimally invasive advantages, it lacks sufficient resistance to vertical shear forces and is less effective in managing femoral neck fractures in young and middle-aged adults, particularly in Pauwels type III fractures, which are associated with a high failure rate (5). Although DHS offers better stability, it fails to meet the demand for minimally invasive treatment due to its high surgical trauma, complex instrumentation, and large volume of internal fixation (6). Therefore, there is an urgent clinical need for an internal fixation system that not only provides adequate stability but also offers the advantage of being minimally invasive.

In recent years, the femoral neck power cross-nailing system (FNS) has emerged as a new type of internal fixation device. Developed by a team at the AO Hip Fracture Research and Development Center, based at the headquarters of Depuy Synthes in Switzerland, the FNS combines the minimally invasive nature of the CCS with the biomechanical stability of the DHS through the cross-locking mechanism of the power bar and staple (7). As the most widely used internal fixation system for femoral neck fractures in recent years, the FNS offers strong resistance to inversion, rotation, and shear forces. Additionally, the surgical procedure is more convenient and minimally invasive compared to the DHS (8), making it an ideal internal fixation modality for the treatment of femoral neck fractures in young and middle-aged adults. Early biomechanical studies and short-term clinical reports suggest that the FNS offers significant advantages in the management of femoral neck fractures, particularly in maintaining fracture reduction and promoting healing (9). Notably, the paired use of the pre-sliding technique has proven effective in preventing femoral neck shortening in the postoperative period (10, 11). However, with the increasing use of FNS, several new complications have gradually emerged. Recent evidence indicates that FNS treatment may be associated with unique complications, such as fracture shortening, screw cutout, and nerve injury, although the underlying mechanisms remain unclear (12). Furthermore, the steep learning curve associated with the FNS technique and the lack of long-term follow-up data continues to raise concerns regarding its safety and efficacy in clinical practice.

The mechanism of treatment failure with the FNS in current clinical practice has not been systematically analyzed. Previous studies have primarily focused on technical failures, such as nail placement angle and power bar tension. However, there has been limited exploration of the interaction between biomechanical failure and individual patient biological factors, such as bone density and blood supply status (13). This knowledge gap not only restricts the further optimization of FNS techniques but also impedes the development of effective strategies for preventing complications.

This retrospective study analyzed clinical data from 89 young and middle-aged patients with femoral neck fractures who were treated with the FNS at Fuzhou Second General Hospital between September 2019 and June 2021. This study aims to identify the causes of early and intermediate-term complications and failures following FNS fixation, to analyze risk factors associated with these complications, and to investigate the influence of pre-compression technique and screw positioning on postoperative outcomes. Based on these findings, the study further seeks to propose novel strategies for preventing and managing FNS-related complications.

## 2 Method

### 2.1 General information

This study strictly followed the strengthening the reporting of observational studies in epidemiology (STROBE) guidelines and was approved by the Medical Ethics Committee of the Fuzhou Second General Hospital (IRB No. 2021185), adhering to the principles of the 2013 declaration of Helsinki.

**Figure 1:**
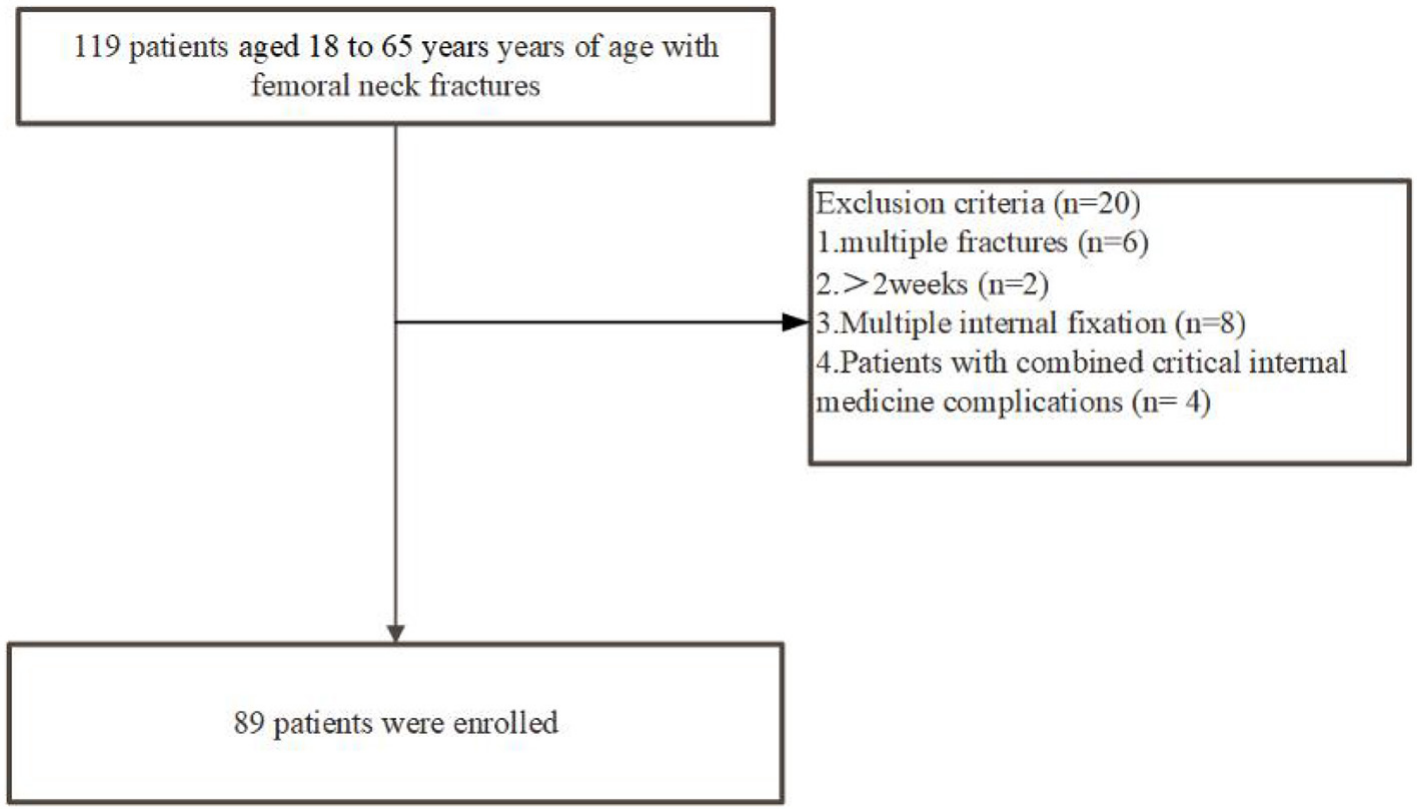
Patient selection flowchart for this study.

This retrospective cohort study analyzed data from 89 patients aged 18 to 65 years with femoral neck fractures who were treated at Fuzhou Second General Hospital between September 2019 and November 2021. Data were collected from the hospital's electronic medical records, medical imaging system, laboratory information system, and follow-up records, with a minimum follow-up period of 6 months. The inclusion and exclusion criteria are summarized in Figure 1.

**Inclusion criteria:** (1) Patients with fresh femoral neck fractures, aged 18–65 years; (2) FNS fixation; (3) Closed reduction; (4) The Garden femoral neck fracture reduction quality assessment method was used to evaluate the reduction outcome; (5) Accurate records of common early and mid-term complications during the operation and follow-up were available.

**Exclusion criteria:** (1) Presence of fractures in other parts of the lower limbs or serious underlying diseases affecting the evaluation of reduction and postoperative outcomes; (2) Pre-existing hip disorders affecting postoperative evaluation; (3) Postoperative follow-up duration < 6 months; (4) Incomplete clinical and imaging data.

### 2.2 Treatment

#### 2.2.1 Surgical method

After the patient was adequately anesthetized using either general anesthesia or intralesional anesthesia, the patient was positioned supine. The healthy side lower limb was fixed on a tripod in an abducted amputation position, while a pillow was placed under the affected side's buttocks to ensure the affected limb was in a neutral and straight position. The femoral vascular alignment of the affected side was marked on the body surface, and the area was routinely sterilized. A sterile towel was placed around the surgical site.

A 2.5 mm Kirschner pin was percutaneously pre-positioned along the anterior superior and posterior superior aspects of the femoral neck under fluoroscopic guidance from a C-arm X-ray machine to prevent rotation. The Kirschner pins were not inserted through the fracture line at this stage. The fracture ends were reduced using conventional methods such as hip flexion/traction, adduction/abduction, and internal rotation. If these manipulations were unsuccessful, a Schanz nail was percutaneously inserted into the lateral aspect of the greater trochanter, with a “T” handle attached. The Schanz nail was then retracted outward and downward along the femoral neck's long axis to unlock the clavicular fracture's end compression and displacement. At the same time, the lower limb was internally rotated by 15°. The traction angle of the Schanz nail was adjusted according to the fracture line alignment, and the fracture was reduced using the “distal to proximal” principle.

If the femoral neck still exhibited anterior angulation deformity on the lateral C-arm X-ray view, a top bar was placed at the outer edge of the femoral artery (aligned with the projection line of the Smith-Peterson incision). The top bar was applied to the distal end of the femoral neck fracture line, and backward pressure was applied to correct the residual anterior angulation and backward inversion of the femoral head. Simultaneously, the assistant assisted in the reduction according to fluoroscopic images, combined with longitudinal traction of the affected limb, internal rotation, internal retraction, or abduction of the lower limb. Once the fracture was satisfactorily reduced, two pre-positioned Kirschner pins were inserted for temporary fixation of the fracture ends, and the Schanz nails were removed.

**Figure 2:**
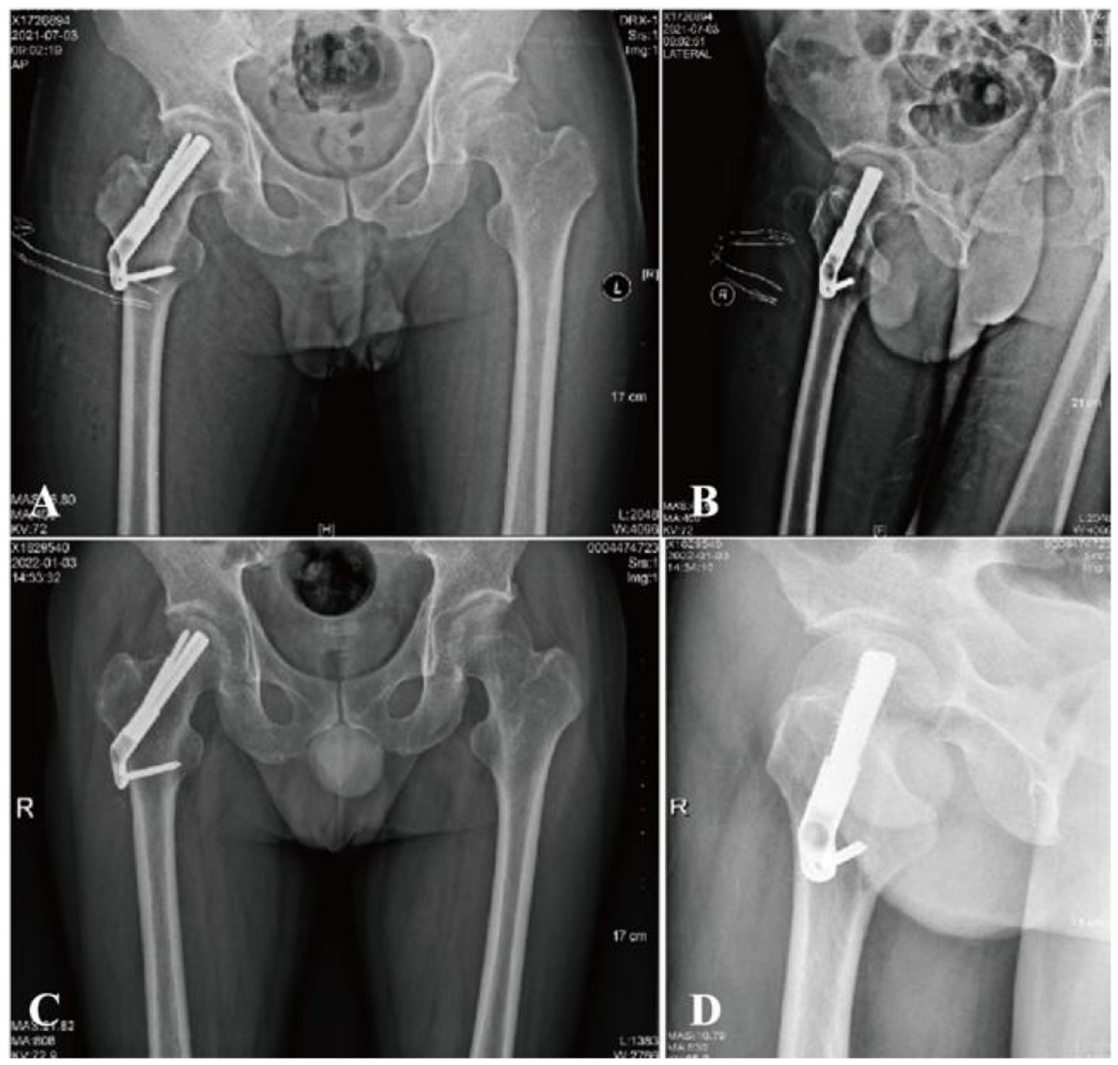
A 36-year-old male with femoral neck fracture. **(A, B)** Postoperative radiographs show good reduction (Garden Index I). **(C, D)** At 6 months, films confirm fracture healing with no shortening or avascular necrosis.

A 5 cm skin incision was made along the proximal thigh at the level of the lateral malleolus, where the longitudinal axis of the femoral stem intersects. A 130° angular guide was used for the insertion of a power bar, positioned in the middle and lower part of the femoral neck in the orthopedic position, or slightly posterior to the femoral neck in the lateral position. The length of the power bar was measured, an opening was created, and the FNS (Johnson & Johnson) power bar was inserted, ensuring the tip of the bar was 5 mm from the subchondral bone of the femoral head. The locking head screw and the anti-retroviral screw were then inserted sequentially. The FNS was positioned satisfactorily on the C-arm X-ray, the two anti-retroviral Kirschner pins were removed, and the wound was irrigated and closed in layers, with a sterile dressing applied (Figure 2).

**Figure 3:**
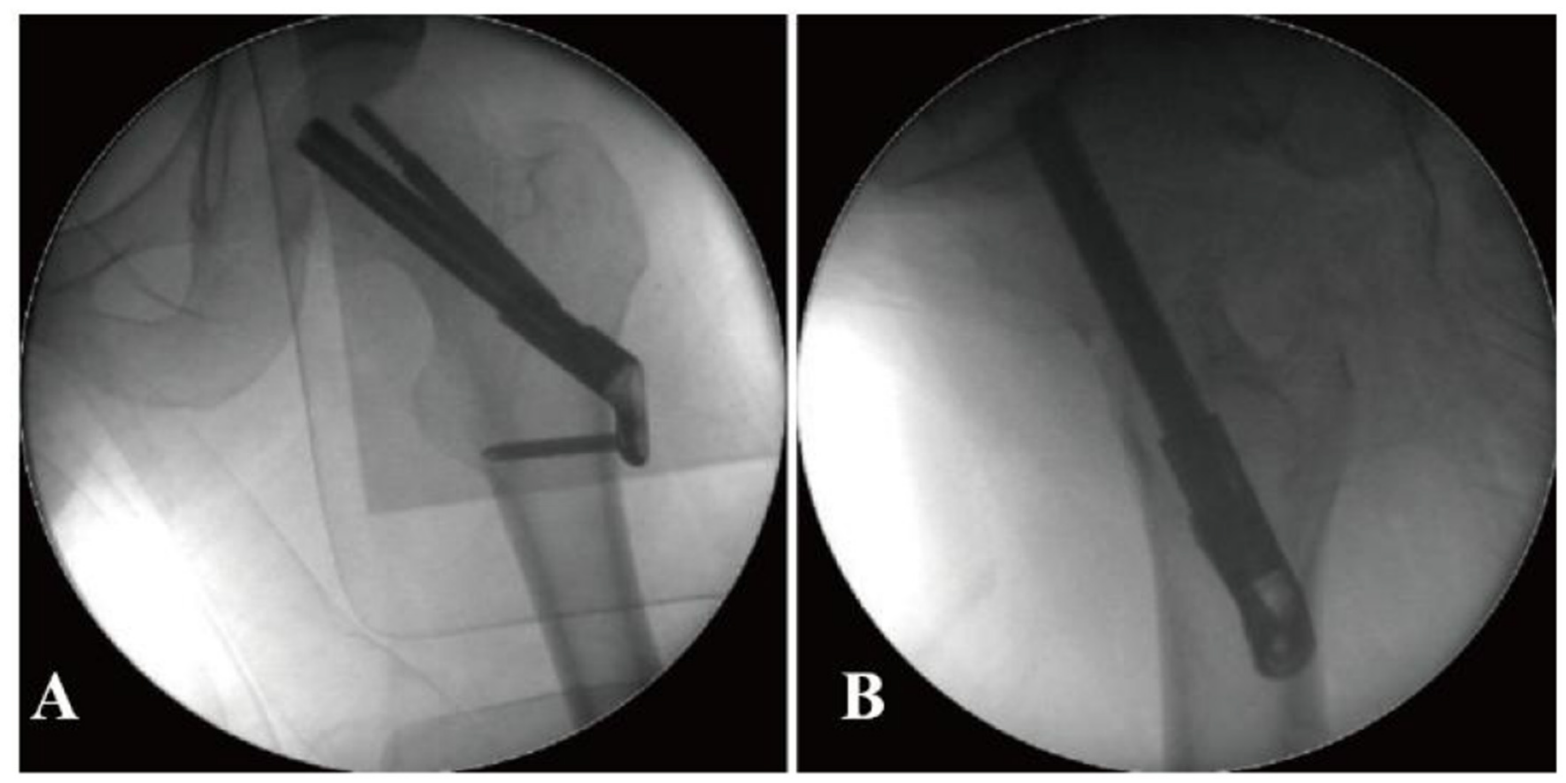
Radiographic and procedural details of FNS implantation in a 41-year-old male with a femoral neck fracture. **(A, B)** Anteroposterior and lateral views show the position of the implant. The tip of the FNS dynamic bolt was placed 5 mm from the femoral subchondral bone, followed by sequential insertion of the locking head screw and the anti-rotation screw. Due to impaction of the posterior cortex, the sliding distance of the FNS was preoperatively adjusted and limited to within 5 mm before final implantation to prevent excessive femoral neck shortening.

Prior to the final implantation of the FNS, a pre-compression technique was employed. This involved adjusting the sliding distance of the implant before its insertion and limiting the sliding distance to within 5 mm. The specific surgical procedure is shown in Figure 3.

#### 2.2.2 Postoperative treatment

On the day of the surgery, a 2.0 g dose of cefazolin was administered intravenously to prevent infection. Low molecular weight heparin sodium was introduced 24 h after surgery to prevent thrombosis. Following hospital discharge, oral anticoagulation therapy was switched to beritol and continued for 35 days postoperatively. Isometric quadriceps contraction training began after the patient regained consciousness from anesthesia. Limited weight-bearing activities on crutches were allowed after postoperative radiographs were taken, with an initial load of 1/3 of the body weight. Straight leg raises and cross-legged rotational movements were prohibited. Full weight-bearing activities were permitted on crutches once bony healing was confirmed.

### 2.3 Observational indicators

Intraoperative and postoperative surgical complications were documented. Regular follow-up radiographs were taken at 1, 3, 6, and 12 months postoperatively, and annually thereafter, to assess the internal fixation failure rate in patients. The degree of femoral neck shortening was evaluated based on the final follow-up.

The quality of fracture reduction was assessed using the Garden's index, which considers two key angles: (1) the angle between the medial edge of the femoral stem and the trabecular axis of the femoral head on the orthopantomogram, and (2) the angle between the femoral head axis and the femoral neck axis on the lateral radiograph. The classification criteria for fracture reduction were as follows:

1) Grade I: 160° on orthopantomogram, 180° on lateral radiographs

2) Grade II: 155° on orthopantomogram, 180° on lateral radiographs

3) Grade III: < 150° on orthopantomogram or >180° on lateral radiographs

4) Grade IV: 150° on orthopantomogram, >180° on lateral radiographs

**Figure 4:**
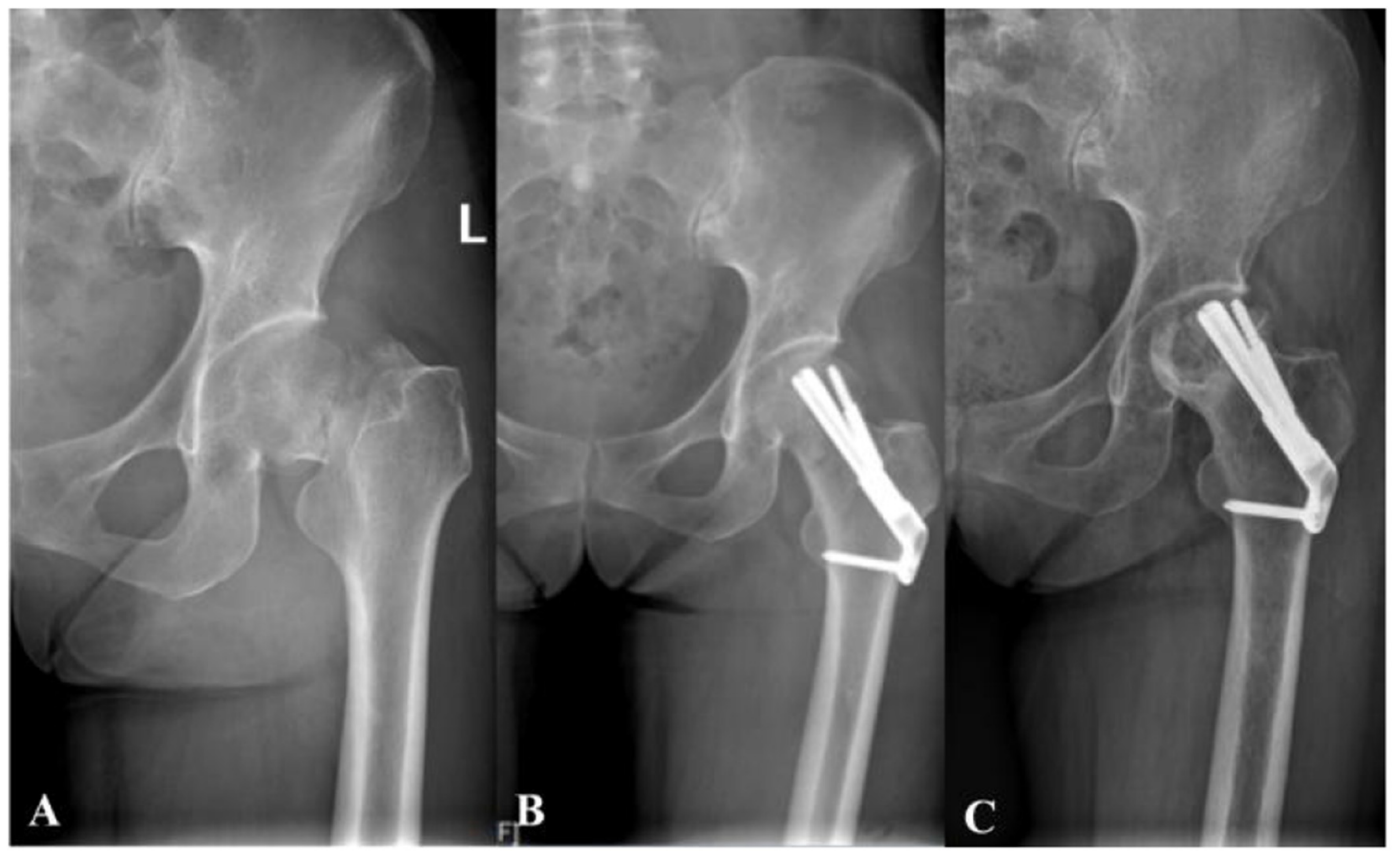
A 38-year-old female with femoral neck fracture. **(A)** Preoperative radiograph shows the initial fracture pattern. **(B)** Immediate postoperative radiograph demonstrates suboptimal positioning of the FNS dynamic screw in the superior region of the femoral head. **(C)** Final follow-up radiograph confirms the development of femoral head osteonecrosis with subsequent collapse.

FNS: On the final follow-up radiograph, the unaffected hip was mirrored to the affected side for comparison. Horizontal shortening (ΔX) was defined as the difference in the horizontal distance between the femoral head centers along the X-axis, while vertical shortening (ΔY) was measured as the vertical distance difference at the base of the femoral heads along the Y-axis. The axial shortening distance (ΔZ) was calculated using the formula: ΔZ = ΔY·sin θ + ΔX·cos θ, where θ represents the angle between the Y-axis and the femoral neck axis. Femoral neck shortening at final follow-up was classified into three categories: none/mild (< 5 mm), moderate (5–10 mm), and severe (>10 mm) (14). Shortening of less than 5 mm is generally attributed to physiologic sliding of the FNS, which promotes compression at the fracture site during healing and is not regarded as a surgical complication (Figure 4).

TAD: The TAD is determined by measuring the distance from the tip of the screw (Tip) to the apex (Apex), which is the intersection extended from the central axis of the femoral head-neck and the articular surface of the femoral head. This measurement is taken in the anteroposterior (Xap) and lateral (Xlat) views. The magnification factor between the actual bolt width (Dtrue) and the bolt width measured on the X-ray (Dap and Dlat) needs to be corrected. The formula for calculating the TAD value is: TAD = Xap × (Dtrue/Dap) + Xlat × (Dtrue/Dlat). In clinical practice, a TAD greater than 25 mm is considered to be associated with an increased risk of complications.

Internal fixation failure: Any breakage of internal fixation components, cut-out of the power bar or screw, or bone nonunion was classified as internal fixation failure.

Power bar position: In this study, the position of the FNS power bar was classified as “superior” if it was located entirely above the midline of the femoral neck. A position at or below the midline was considered acceptable.

### 2.4 Statistical analysis

All statistical analyses were performed with IBM SPSS Statistics 29.0. Continuous variables were first tested for normality using the Shapiro–Wilk test. Normally distributed data are presented as mean ± SD, whereas non-normally distributed data are presented as median (P25, P75). Comparisons between two groups were conducted with the Mann–Whitney U test. Categorical variables are expressed as frequencies and percentages. Fisher's exact test was used when the expected count in any cell was ≤ 5; otherwise, Pearson's χ^2^ test was applied. A two-tailed α level of 0.05 was adopted; *P* ≤ 0.05 was considered statistically significant.

## 3 Result

### 3.1 Baseline characteristics

Initially, 119 patients with femoral neck fractures were enrolled; 20 were excluded during follow-up, leaving 89 for the final analysis. Twelve patients (13.5%) were assigned to the complication group and 77 (86.5%) to the non-complication group. The cohort comprised 42 men (47.2%) and 47 women (52.8%) with a mean age of 39.8 ± 8.9 years. No significant between-group differences were observed in age, sex, affected side, BMI, or smoking/alcohol history (*P* > 0.05), indicating balanced baseline characteristics. The overall mean fracture-healing time was 10.06 ± 0.52 weeks.

Regarding fracture morphology, displaced fractures accounted for 55 cases (61.8%) and non-displaced for 34 (38.2%), with no significant inter-group difference. Medial cortical comminution was present in 10 patients (83.3%) in the complication group vs. 23 (29.9%) in the non-complication group, a difference that was statistically significant (Table 1).

**Table 1:** Baseline characteristics of patients with femoral neck fractures.

**Variables**	**Total (*n* = 89)**	**Non-complications (*n* = 77)**	**Complications (*n* = 12)**	**Statistic**	***P***
Age, Mean ± SD	39.80 ± 8.93	39.86 ± 8.95	39.42 ± 9.13	*t* =0.16	0.875
**Gender**, ***n*** **(%)**				*χ^2^* = 0.04	0.834
Female	47 (52.81)	41 (53.25)	6 (50.00)		
Male	42 (47.19)	36 (46.75)	6 (50.00)		
**Side**, ***n*** **(%)**				*χ^2^* = 0.25	0.620
Left	43 (48.31)	38 (49.35)	5 (41.67)		
Right	46 (51.69)	39 (50.65)	7 (58.33)		
**Alcoholist**, ***n*** **(%)**				*χ^2^* = 0.50	0.481
No	51 (57.30)	43 (55.84)	8 (66.67)		
Yes	38 (42.70)	34 (44.16)	4 (33.33)		
**Smoking**, ***n*** **(%)**				*χ^2^* = 0.00	1.000
No	57 (64.04)	49 (63.64)	8 (66.67)		
Yes	32 (35.96)	28 (36.36)	4 (33.33)		
**Medial cortical comminution**, ***n*** **(%)**				*χ^2^* = 10.53	0.001
No	56 (62.92)	54 (70.13)	2 (16.67)		
Yes	33 (37.08)	23 (29.87)	10 (83.33)		
**Injury mechanism**, ***n*** **(%)**				*χ^2^* = 1.78	0.183
Low-energy trauma	38 (42.70)	35 (45.45)	3 (25.00)		
High-energy trauma	51 (57.30)	42 (54.55)	9 (75.00)		
**Fracture type (Garden)**, ***n*** **(%)**				*χ^2^* = 0.48	0.489
Non-displaced	34 (38.20)	31 (40.26)	3 (25.00)		
Displaced	55 (61.80)	46 (59.74)	9 (75.00)		

t, t-test; Z, Mann-Whitney test; χ^2^, Chi-square test; -, Fisher exact. SD, standard deviation; M, Median; Q1, 1st Quartile; Q3, 3st Quartile.

### 3.2 Surgical data

Intraoperative data are shown in Table 2. The mean operative time for all patients was 0.94 ± 0.39 min, the preoperative waiting time was 2 (1–3) days, and the estimated blood loss was 50 (30–80) ml; none of these parameters differed significantly between the two groups. TAD ≥ 20° was observed in 9 (75.0%) patients in the complication group vs. 17 (22.1%) in the non-complication group, with a statistically significant difference (*P* < 0.001). Regarding reduction quality, Garden grade III was found in 3 (25.0%) vs. 8 (10.4%) patients, and Garden grade IV in 6 (50.0%) vs. 3 (3.9%) patients, respectively; both comparisons were statistically significant (*P* < 0.001). Superior power-bar placement was recorded in 4 (33.3%) vs. 63 (81.8%) patients, showing a significant difference (*P* < 0.001). The pre-sliding technique was used in 2 (16.7%) vs. 36 (46.8%) patients, with a statistically significant difference (*P* = 0.050).

**Table 2:** Surgical data of patients treated with femoral neck system (FNS).

**Variables**	**Total (*n* = 89)**	**Non-complications (*n* = 77)**	**Complications (*n* = 12)**	**Statistic**	***P***
Surgical time, Mean ± SD	0.94 ± 0.39	0.96 ± 0.41	0.80 ± 0.23	*t* = 1.33	0.187
Intraoperative blood Loss, M (Q1, Q3)	50.00 (30.00, 80.00)	50.00 (30.00, 80.00)	45.00 (20.00, 72.50)	*Z* = −0.10	0.923
Preoperative waiting time, M (Q1, Q3)	2.00 (1.00, 3.00)	2.00 (1.00, 3.00)	2.00 (2.00, 3.00)	*Z* = −0.66	0.507
**TAD**, ***n*** **(%)**				*χ^2^* = 11.62	< 0.001
< 25°	63 (70.79)	60 (77.92)	3 (25.00)		
≥25°	26 (29.21)	17 (22.08)	9 (75.00)		
**Reduction quality (garden index)**, ***n*** **(%)**				–	< 0.001
I	39 (43.82)	38 (49.35)	1 (8.33)		
II	30 (33.71)	28 (36.36)	2 (16.67)		
III	11 (12.36)	8 (10.39)	3 (25.00)		
IV	9 (10.11)	3 (3.90)	6 (50.00)		
**Power bar position**, ***n*** **(%)**				*χ^2^* = 10.64	0.001
Superior displacement	67 (75.28)	63 (81.82)	4 (33.33)		
Well displacement	22 (24.72)	14 (18.18)	8 (66.67)		
**Pre-compression technique**, ***n*** **(%)**				*χ^2^* = 3.84	0.050
No	51 (57.30)	41 (53.25)	10 (83.33)		
Yes	38 (42.70)	36 (46.75)	2 (16.67)		

t, t-test; Z, Mann-Whitney test; χ^2^, Chi-square test; -, Fisher exact. SD, standard deviation; M, Median; Q1, 1st Quartile; Q3, 3st Quartile.

### 3.3 Follow-up outcomes and complications

Follow-up data showed (Table 3): There was no statistically significant difference in postoperative Harris scores between the two groups (26.9 ± 3.3 vs. 27.1 ± 3.6) (*P* = 0.847); There was no statistically significant difference in Harris scores at 3 months postoperatively between the two groups (56.9 ± 2.6 vs. 56.3 ± 2.6) (*P* = 0.394); The difference in Harris scores between the two groups at 6 months postoperatively was statistically significant (71.3 ± 2.6 vs. 79.6 ± 2.9) (*P* < 0.001); The difference in Harris scores between the two groups at 1 year postoperatively was statistically significant (77.2 ± 4.0 vs. 87.3 ± 3.3) (*P* < 0.001).

**Table 3:** Follow-up outcomes and complications of patients after FNS treatment.

**Variables**	**Total (*n* = 89)**	**Non-complications (*n* = 77)**	**Complications (*n* = 12)**	**Statistic**	***P***
Postoperative Harris score, Mean ± SD	26.91 ± 3.32	26.88 ± 3.30	27.08 ± 3.58	*t* = −0.19	0.847
Three month Harris score, Mean ± SD	56.92 ± 2.55	57.01 ± 2.55	56.33 ± 2.64	*t* = 0.86	0.394
Six month Harris score, Mean ± SD	78.52 ± 4.03	79.64 ± 2.89	71.33 ± 2.64	*t* = 9.36	< 0.001
Final Harris score, Mean ± SD	85.90 ± 4.86	87.26 ± 3.34	77.17 ± 4.02	*t* = 9.48	< 0.001
**Complication**, ***n*** **(%)**				–	< 0.001
No	77 (86.52)	77 (100.00)	0 (0.00)		
Femoral neck shortening	9 (10.11)	0 (0.00)	9 (75.00)		
Lateral femoral cutaneous nerve injury	1 (1.12)	0 (0.00)	1 (8.33)		
Internal fixation failure	2 (2.25)	0 (0.00)	2 (16.67)		

t, t-test; Z, Mann-Whitney test; χ^2^, Chi-square test; -, Fisher exact. SD, standard deviation; M, Median; Q1, 1st Quartile; Q3, 3st Quartile.

Complication data showed: A total of 12 patients (13.5%) experienced complications, and 77 patients (86.5%) did not experience complications (Table 3). The incidence of femoral neck shortening was 10.1% (9 cases); the incidence of lateral femoral cutaneous nerve injury was 1.1% (1 case), and the incidence of internal fixation failure was 2.2% (2 cases).

### 3.4 Pre-compression technique

Patients were divided into a pre-compression group (*n* = 38) and a no-pre-compression group (*n* = 51) according to whether the pre-compression technique was used (Table 4). There were no statistically significant differences between the two groups in baseline data such as age (*t* = −0.59, *p* = 0.556), gender (χ^2^ = 0.69, *p* = 0.407), or affected side (χ^2^ = 0.49, *p* = 0.482), indicating balanced comparability. Fracture morphology, including fracture type (χ^2^ = 0.05, *p* = 0.831) and medial cortical comminution (χ^2^ = 0.86, *p* = 0.354), also showed no significant differences. Surgical-related variables—operative time (*t* = 0.23, *p* = 0.816), preoperative waiting time (*Z* = −1.10, *p* = 0.273), intraoperative blood loss (*Z* = −0.45, *p* = 0.652), reduction quality (*p* = 0.962), TAD (χ^2^ = 0.98, *p* = 0.322), and power-bar position (χ^2^ = 0.09, *p* = 0.763)—did not differ significantly between groups.

**Table 4:** Pre-compression technique impact on characteristics and outcomes.

**Variables**	**Total (*n* = 89)**	**No (*n* = 51)**	**Yes (*n* = 38)**	**Statistic**	***P***
Age, Mean ± SD	39.80 ± 8.93	39.31 ± 8.69	40.45 ± 9.31	*t* = −0.59	0.556
Surgical time, Mean ± SD	0.94 ± 0.39	0.94 ± 0.44	0.92 ± 0.32	*t* = 0.23	0.816
Postoperative Harris score, Mean ± SD	26.91 ± 3.32	26.94 ± 3.04	26.87 ± 3.70	*t* = 0.10	0.919
Three month Harris score, Mean ± SD	56.92 ± 2.55	56.98 ± 2.37	56.84 ± 2.81	*t* = 0.25	0.802
Six month Harris score, Mean ± SD	78.52 ± 4.03	77.45 ± 4.63	79.95 ± 2.43	*t* = −3.29	0.001
Final Harris score, Mean ± SD	85.90 ± 4.86	84.65 ± 5.04	87.58 ± 4.10	*t* = −2.93	0.004
Intraoperative Blood Loss, M (Q1, Q3)	50.00 (30.00, 80.00)	50.00 (20.00, 80.00)	50.00 (40.00, 70.00)	*Z* = −0.45	0.652
Preoperative waiting time, M (Q1, Q3)	2.00 (1.00, 3.00)	2.00 (1.00, 3.00)	2.00 (2.00, 3.00)	*Z* = −1.10	0.273
**Gender**, ***n*** **(%)**				*χ^2^* = 0.69	0.407
Female	47 (52.81)	25 (49.02)	22 (57.89)		
Male	42 (47.19)	26 (50.98)	16 (42.11)		
**Side**, ***n*** **(%)**				*χ^2^* = 0.49	0.482
Left	43 (48.31)	23 (45.10)	20 (52.63)		
Right	46 (51.69)	28 (54.90)	18 (47.37)		
**Alcoholist**, ***n*** **(%)**				*χ^2^* = 0.93	0.335
No	51 (57.30)	27 (52.94)	24 (63.16)		
Yes	38 (42.70)	24 (47.06)	14 (36.84)		
**Smoking**, ***n*** **(%)**				*χ^2^* = 5.68	0.088
No	57 (64.04)	38 (74.51)	19 (50.00)		
Yes	32 (35.96)	13 (25.49)	19 (50.00)		
**TAD**, ***n*** **(%)**				*χ^2^* = 0.98	0.322
< 25°	63 (70.79)	34 (66.67)	29 (76.32)		
≥25°	26 (29.21)	17 (33.33)	9 (23.68)		
**Medial cortical comminution**, ***n*** **(%)**				*χ^2^* = 0.86	0.354
No	56 (62.92)	30 (58.82)	26 (68.42)		
Yes	33 (37.08)	21 (41.18)	12 (31.58)		
**Injury mechanism**, ***n*** **(%)**				*χ^2^* = 0.93	0.335
Low-energy trauma	38 (42.70)	24 (47.06)	14 (36.84)		
High-energy trauma	51 (57.30)	27 (52.94)	24 (63.16)		
**Reduction quality (garden index)**, ***n*** **(%)**				–	0.962
I	39 (43.82)	22 (43.14)	17 (44.74)		
II	30 (33.71)	17 (33.33)	13 (34.21)		
III	11 (12.36)	6 (11.76)	5 (13.16)		
IV	9 (10.11)	6 (11.76)	3 (7.89)		
**Fracture type (Garden)**, ***n*** **(%)**				*χ^2^* = 0.05	0.831
Non-displaced	34 (38.20)	19 (37.25)	15 (39.47)		
Displaced	55 (61.80)	32 (62.75)	23 (60.53)		
**Power bar position**, ***n*** **(%)**				*χ^2^* = 0.09	0.763
Superior displacement	67 (75.28)	39 (76.47)	28 (73.68)		
Well displacement	22 (24.72)	12 (23.53)	10 (26.32)		
**Complication**, ***n*** **(%)**				–	0.007
No	77 (86.52)	41 (80.39)	36 (94.74)		
Femoral neck shortening	9 (10.11)	9 (17.65)	0 (0.00)		
Lateral femoral cutaneous nerve injury	1 (1.12)	0 (0.00)	1 (2.63)		
Internal fixation failure	2 (2.25)	1 (1.96)	1 (2.63)		

t, t-test; Z, Mann-Whitney test; χ^2^, Chi-square test; -, Fisher exact. SD, standard deviation; M, Median; Q1, 1st Quartile; Q3, 3st Quartile.

Complication data: In the pre-compression group, femoral-neck shortening occurred in 0 cases (0%), lateral femoral cutaneous nerve injury in 1 case (2.6%), and internal-fixation failure in 1 case (2.6%). In the no-pre-compression group, femoral-neck shortening occurred in 9 cases (17.6%), lateral femoral cutaneous nerve injury in 0 cases (0%), and internal-fixation failure in 1 case (2.0%).

Follow-up Harris scores: No significant inter-group differences were observed postoperatively (26.9 ± 3.7 vs. 26.9 ± 3.0, *P* = 0.919) or at 3 months (56.8 ± 2.8 vs. 57.0 ± 2.3, *P* = 0.802); however, differences were significant at 6 months (79.9 ± 2.4 vs. 77.5 ± 4.6, *P* = 0.001) and at 1 year (87.6 ± 4.1 vs. 84.7 ± 5.0, *P* = 0.004).

### 3.5 Risk-factor analysis

Univariate analysis identified five potential risk factors: TAD (*P* = 0.001), medial cortical comminution (*P* = 0.002), injury mechanism (*P* = 0.194), reduction quality (*P* < 0.001), and power bar position (*P* = 0.001; Table 5). Multivariate logistic regression analysis showed that TAD (OR 10.59, 95% CI 2.58–43.51, *P* = 0.001), medial cortical comminution (OR 11.74, 95% CI 2.38–57.84, *P* = 0.002), reduction quality (III) (OR 14.25, 95% CI 1.31–155.23, *P* = 0.029), reduction quality (IV) (OR 76.00, 95% CI 6.75–855.89, *P* < 0.001), and power bar position (well displacement) (OR 9.00, 95% CI 2.37–34.12, *P* = 0.001) were all independent significant predictors of postoperative complications (Table 6).

**Table 5:** Univariate analysis of risk factors for postoperative complications.

**Variable**	***N***	**OR**	**95%CI**	***P-value***
Age	89.0	0.994	[0.928–1.065]	0.873
BMI	89.0	0.663	[0.408–1.077]	0.097
Surgical time	89.0	0.247	[0.03–2.021]	0.192
Preoperative waiting time	89.0	1.124	[0.629–2.007]	0.693
Intraoperative blood loss	89.0	0.999	[0.978–1.02]	0.900
**Gender**				
Female	47.0			
Male	42.0	1.139	[0.337–3.846]	0.834
**Side**				
Left	43.0			
Right	46.0	1.364	[0.398–4.674]	0.621
**Alcoholist**				
No	51.0			
Yes	38.0	0.632	[0.176–2.278]	0.483
**Smoking**				
No	57.0			
Yes	32.0	0.875	[0.242–3.169]	0.839
**TAD**				
< 25°	63.0			
≥25°	26.0	10.588	[2.577–43.511]	0.001
Medial cortical comminution				
No	56.0			
Yes	33.0	11.739	[2.383–57.836]	0.002

Low-energy trauma	38.0			
High-energy trauma	51.0	2.500	[0.628–9.952]	0.194
**Reduction quality**				
I	39.0			
II	30.0	2.714	[0.234–31.441]	0.424
III	11.0	14.250	[1.308–155.226]	0.029
IV	9.0	76.000	[6.749–855.889]	0.000
**Fracture type (Garden)**				
Non-displaced	34.0			
Displaced	55.0	2.022	[0.507–8.066]	0.319
**Power bar position**				
Superior displacement	67.0			
Well displacement	22.0	9.000	[2.374–34.119]	0.001
**Pre-compression technique**				
No	51.0			
Yes	38.0	0.228	[0.047–1.109]	0.067

**Table 6:** Multivariate analysis of risk factors for postoperative complications.

**Characteristic**	**Descript**	***P* (multivariable)**	***OR* (95% CI) (multivariable)**
TAD	< 25°		
	≥25°	0.034	1.83 (1.25–5.35)
Medial cortical comminution	No		
	Yes	0.01	4.24 (2.53–8.15)
Reduction quality (garden index)	I		
	II	0.165	9.67 (0.39–23.39)
	III	0.011	1.34 (1.03–11.64)
	IV	0.003	2.89 (1.86–15.07)
Power bar position	Superior displacement		
	Well displacement	0.045	2.90 (1.32–5.46)

## 4 Discussion

The treatment of femoral neck fractures in young and middle-aged adults remains a significant challenge, with no consensus on the optimal fixation method (15, 16) A meta-analysis by Slobogean et al. (17), which included 41 studies covering a total of 1,558 femoral neck fractures in young and middle-aged adults, found that the postoperative rate of femoral head collapse and necrosis was 14.3%, while the rate of internal fixation failure was as high as 9.3%. Most researchers agree that the primary factors influencing femoral head necrosis are the degree of initial fracture displacement, the quality of fracture reduction, and the method of internal fixation (18, 19). Since the degree of initial fracture displacement is an uncontrollable factor, enhancing the quality of reduction and optimizing the fixation technique has become critical in reducing the incidence of femoral head necrosis (20, 21). Since 2019, femoral neck screw (FNS) fixation has been widely adopted in China, demonstrating good short-term efficacy due to its combination of the minimally invasive advantages of the hollow screw system and the stability of DHS-like internal fixation. However, complications can still occur regardless of the type of internal fixation, including FNS fixation, in the treatment of femoral neck fractures (22, 23). In this study, 23 out of 89 patients treated with FNS developed complications of varying degrees, resulting in an incidence rate of 25.84%. Among these complications, femoral neck shortening was the most common (24). The incidence of moderate shortening in femoral neck fractures treated with FNS was 6.74%, while the incidence of severe shortening was 3.37%. These findings suggest that while FNS offers certain advantages in terms of stability, it still requires further optimization.

The mechanism of femoral neck shortening can be categorized into two types: shortening due to poor reduction and shortening despite good reduction, often accompanied by cortical comminution. In this study, the two cases of severe shortening were associated with poor reduction and improper proximal internal fixation positioning, which led to inadequate cortical support at the fracture site. This, in turn, prevented the FNS power bar from counteracting shear and inversion stresses, potentially contributing to the severe shortening or even failure of the femoral neck (25). Regardless of the type of fixation used, good reduction remains the fundamental basis for the successful treatment of femoral neck fractures. As early as 1971, Garden RS emphasized that the quality of reduction is the most crucial factor in minimizing complications associated with femoral neck fractures (26, 27). Moreover, among the seven patients with good reduction, six experienced moderate shortening, and one had severe shortening, which may be linked to the presence of comminuted or free bone fragments at the fracture site (e.g., in the case of severe shortening). Even if the fracture is properly repositioned, load sharing is often not achieved at the fracture site (28, 29), and during weight-bearing activities, the fracture may fail to withstand the load, leading to further shortening. The 20-mm sliding compression reserved for the FNS may be insufficient to support the weight-bearing load at the fracture site, particularly in cases of comminuted or deficient cortical bone. This results in compressive stresses being transmitted to the internal fixation, which may cause excessive sliding of the power bar until it reaches a point where it can sufficiently support the compressive forces, stabilizing the shortening of the femoral neck. The mechanism of power bar cutout is also closely related to positional deviation. Both cases of power rod cutouts in this study were associated with improper positioning of the power bar. Studies by Goffin et al. (30), Kuzyk et al. (31), and others have demonstrated that hip screws are most prone to cutout when positioned in the mid-inferior or inferior-posterior regions of the femoral head and neck. Biomechanical and clinical studies on the positioning of internal fixation in hip fractures, including intertrochanteric fractures, have reached similar conclusions (32–35). Intraoperative visual bias may contribute to the superior placement of the power bar. Although the guide pin appears to be centered on the femoral head and neck in the C-arm orthopantomography, the actual placement of the power bar may be more superior than intended. During surgery, the FNS guide pin is typically placed in the mid-lower femoral neck position laterally, with the tip of the guide pin directed toward the subchondral bone of the femoral head. This ensures that the power bar is positioned in the lower mid-femoral neck region, laterally centered in the head and neck or slightly posterior to it. The additional benefit of placing the power bar inferiorly is that temporary fixation with a Kirschner pin does not obstruct or interfere with the placement of the power bar or anti-rotational screw. The power bar should be tapped gently to avoid penetrating the subchondral bone. To minimize this risk, it is recommended to use a standardized biplane fluoroscopy procedure (orthogonal and lateral views) combined with guided pin placement.

Although the technique for femoral neck fracture reduction has advanced in recent years, certain cases still present challenges, such as difficult-to-reduce femoral neck fractures. Subtalar-type fractures with complete displacement of the femoral head and neck may be effectively reduced using the three-dimensional interactive technique. However, this technique carries the risk of damaging the lateral femoral cutaneous nerve during Kirschner pin insertion into the femoral head, a complication observed in this study. Clinically, some Garden type III and IV fractures exhibit a bird's beak shape, making satisfactory reduction difficult even with the three-dimensional interactive technique. In this study, we employed the “dynamic universal traction-roofing technique,” a non-traction bed method that utilizes the Schanz nail and top bar as key components to achieve satisfactory reduction. However, during the reduction process, the Schanz nail may become loosened or displaced if handled improperly. Since the cases in this study were distributed across different treatment groups, the quality of reduction varied depending on the specific technique employed.

Regarding the Kirschner-wire breakage: the fragment was generated when the step drill inadvertently engaged the temporary 2.5 mm anti-rotation pin; the reaming torque exceeded the wire's bending limit and sheared it at the cortical entry point. This technical mishap is uncommon and can be prevented by maintaining ≥15 mm clearance between any provisional wire and the drill path, upgrading to a 3.0 mm wire when space is limited, and reaming slowly under live fluoroscopy.

In light of the aforementioned issues, this study proposes the following directions for technical improvement: First, optimizing the quality of repositioning is crucial. The use of the “non-traction bed dynamic universal traction-rope push reset technique” can enhance the quality of resetting; however, care must be taken to prevent the loosening of the Schanz nails due to excessive force. It is recommended to employ self-tapping Schanz nails or bone cement to improve anchoring strength. Second, the power bar should be positioned orthogonally at the middle and lower part of the femoral neck and laterally at the mid or slightly posterior part of the head and neck, to avoid excessive tapping, which could result in subchondral bone penetration (32, 35). Finally, in the case of comminuted fractures, the FNS power bar's sliding compression distance can be pre-set. It is advised to use a power bar that is longer than the measured value and to incorporate a pre-compression mechanism, retracting the power bar to reduce the sliding space. Additionally, delaying or limiting weight-bearing in the postoperative phase may help. These improvements are expected to reduce complication rates and enhance the success of FNS treatment.

This study also has several limitations. First, the retrospective design may have introduced selection bias (e.g., open fractures and pathologic fractures were excluded). Second, the follow-up period was relatively short, and as a result, mid-and long-term complications (e.g., femoral head necrosis) may not have been captured. These complications would require long-term follow-up, as the onset of necrosis often occurs after more than 2 years. Additionally, the variability in repositioning techniques may have influenced the results. The significant impact of operator experience on complication rates suggests that future studies should incorporate subgroup analyses based on the years of experience of the operators and their associated complication rates.

In summary, FNS combines the minimally invasive advantages of hollow screws with the stability of DHS, positioning it as a promising fixation method for femoral neck fractures in young and middle-aged adults. However, a standardized surgical approach and high-quality reduction are crucial for minimizing complications. Based on the findings of this study, we propose a standardized operative framework for FNS, which includes: prioritizing reduction (Garden's alignment index ≥160°), low placement of power rods (TAD < 25 mm), and pre-compression management of comminuted fractures. A multicenter prospective study is necessary to validate the effectiveness of this approach and to further optimize the clinical application of FNS.

## 5 Conclusion

The study highlights that precise surgical technique is crucial for minimizing complications. It is recommended that the power bar be placed in the lower and middle parts of the femoral neck during surgery and that a pre-compression technique be employed to reduce the risk of shortening. Additionally, optimizing repositioning techniques (e.g., the “non-traction bed dynamic universal traction-overhead thrust repositioning technique”) and implementing strict postoperative weight-bearing management are essential to improving outcomes. Although FNS has demonstrated good short-term efficacy, its long-term safety and effectiveness require validation through multicenter, prospective studies with extended follow-up periods to overcome the limitations of selection bias and the short follow-up time inherent in retrospective studies. This study proposes a standardized surgical framework that prioritizes ensuring the quality of reduction, precise positioning of power rods, and individualized management strategies for comminuted fractures, all aimed at reducing complications and improving functional recovery in patients.

## Data Availability

The original contributions presented in the study are included in the article/supplementary material, further inquiries can be directed to the corresponding authors.
